# Impact of connective tissue disease on the surgical outcomes of aortic dissection in patients with cystic medial necrosis

**DOI:** 10.1186/s13019-017-0663-8

**Published:** 2017-11-23

**Authors:** Toshiki Fujiyoshi, Kenji Minatoya, Yoshihiko Ikeda, Hatsue Ishibashi-Ueda, Takayuki Morisaki, Hiroko Morisaki, Hitoshi Ogino

**Affiliations:** 1 Departments of Cardiovascular Surgery, National Cerebral and Cardiovascular Center, Osaka, Japan; 2 Departments of Pathology, National Cerebral and Cardiovascular Center, Osaka, Japan; 3 Departments of Bioscience and Genetics, National Cerebral and Cardiovascular Center, Osaka, Japan; 4 Cardiovascular Surgery, Tokyo Medical University, 6-7-1 Nishishinjuku Shinjuku-ku Tokyo, 160, Tokyo, –0023 Japan

**Keywords:** Aortic dissection, Surgery, Genetically diagnosed connective tissue disease, Cystic medial necrosis

## Abstract

**Background:**

A retrospective analysis was performed to determine the impact of genetically diagnosed connective tissue disease (CTD) on the early and late outcomes of surgical treatment for aortic dissection in patients having aortic pathology associated with cystic medial necrosis (CMN).

**Methods:**

Between 2003 and 2013, a total of 43 patients (37 ± 12.8 years old, 23 men, 20 women) who had undergone surgery for aortic dissection associated with CMN in the aortic wall underwent genetic examinations. Subsequently, there were 30 patients with CTD (CTD group) and 13 without CTD (non-CTD group).

**Results:**

There were no early or late deaths (the follow-up rate was 100% for 57.1 ± 43.0 months). The median age was significantly lower in the CTD group (*p* = 0.030). The rate of elastic fiber loss was significantly higher in the CTD group (*p* = 0.014). In the long-term follow-up, there were no significant differences in the incidences of re-dissection (*p* = 0.332). However, re-operations were required more frequently in the CTD group (*p* = 0.037).

**Conclusions:**

In patients with CTD as well as CMN, the onset of aortic dissection tends to be earlier, which would result in higher rates of re-operation, compared with the non-CTD group. Closer and stricter follow-up with medication and adequate surgical treatments with appropriate timing are mandatory for such high-risk patients.

## Background

Cystic medial necrosis (CMN) or degeneration is found in surgical specimens of aortic dissection, and tends to be associated with higher risks of various aortic complications including aortic dissection and dilatation [1–3]. In addition, CMN is also considered as one of the histological markers for connective tissue diseases (CTD), including Marfan syndrome, Loeys-Dietz syndrome, and Ehlers-Danlos syndrome. Although some differences are found in the degree of loss of elastic fibers, there are no significant differences in the histopathological patterns within the aortic wall between CTD and non-CTD patients, especially in patients who are of advanced age and have systemic hypertension for a long time [4, 5]. Further genetic examinations are then highly recommended to diagnose CTD more precisely. However, for every patient with aortic dissection, there are difficulties in performing routine genetic examinations which are generally carried out when association with CTD is suspected, based on bodily features and family histories. On the other hand, there have been few published studies looking at the adverse effects and relationships of CTD and CMN to the surgical outcomes of aortic dissection. Under these circumstances, we wanted to determine the impact of genetically diagnosed CTD on the early and late outcomes of surgical treatment for aortic dissection in patients having pathologies associated with CMN.

## Methods

### Patient profiles

We reviewed our institutional database to identify patients who underwent initial surgery for aortic dissection between April 2003 and March 2013 at the National Cerebral and Cardiovascular Center, Osaka, Japan. A total of 321 patients underwent initial surgery for aortic dissection and 298 patients (64.0 ± 15.7 years old, 56% male) underwent postoperative pathological examinations of the surgical specimen during this period, in which CMN was present in 141 patients (47.3%). Of them, 43 patients (37.0 ± 12.8 years old, 53% male) subsequently underwent genetic examinations. Our criteria for genetic examination were as follows: (1) patients with bodily features consistent with the current Ghent criteria, (2) young patients under 50 years of age, and (3) patients with family history of aortic diseases. This study was approved by the National Cerebral and Cardiovascular Center of Japan Institutional Review Board waived the need for individual patient consent under the provisions for deidentified human subject and quality improvement research.

The patient characteristics are shown in Table 1. Thirty patients (69.8%, CTD group) were diagnosed genetically as having CTD, whereas the remaining 13 patients (30.2%, non-CTD group) did not have any genetic disorders. The details of genetic disorders are shown in Table 2. Preoperative variables were compared between the CTD and non-CTD groups (Table 3). The median age at the onset of aortic dissection was significantly lower in the CTD group, namely 33.5 years in the CTD group vs. 42.0 years in the non-CTD (*p* = 0.030). In addition, there were differences in the Stanford classification of aortic dissection (*p* = 0.015) and the stages of aortic dissection (*p* = 0.009) between the two patient groups. In the CTD group, there were type A aortic dissections in 11 patients (37%, 7 acute and 4 chronic) and type B aortic dissections in 19 patients (63%, 1 acute and 18 chronic). In the non-CTD group, there were type A aortic dissections in 10 patients (77%, 9 acute and 1 chronic) and type B in 3 patients (23%, 3 chronic). The incidence of type B aortic dissection was higher in the CTD group. Two patients in the CTD group developed three-channel aortic dissection, although it was not found in the non-CTD group. The incidence of family history of aortic dissection was significantly higher in the CTD group (57% vs. 23%, *p* = 0.043). The rate of preoperative renal dysfunction (serum creatinine ≥2.0) was significantly higher in the non-CTD group (*p* = 0.028).

**Table 1:** Patient characteristics

Patient characteristics (*n* = 43)	
Male: Female	23: 20
Mean age at AD (years)	37.0 ± 12.8
Stanford AD classification	
Type A	21 (48.8%)
Type B	22 (51.2%)
Stage of aortic dissection	
Acute	17 (39.5%)
Chronic	26 (60.5%)
Connective tissue disorder^a^ 30 (69.8%)	

*AD* aortic dissection

^a^genetically diagnosed

**Table 2:** Genetic disorders in CTD

Genetic disorders in CTD	Number
*FBN1*	19
*TGFBR2*	4
*ACTA2*	3
*MYH11*	1
*SMAD3*	1
*TGFB2*	1
*COLIAI*	1

*CTD* genetically diagnosed connective tissue disease

**Table 3:** Preoperative variables

Preoperative variables (n = 43)	CTD (*n* = 30)	non-CTD (*n* = 13)	*p* value
Male	14 (46.7%)	9 (69.2%)	0.303
Median age at AD (years [range])	33.5 [19–65]	42.0 [28–71]	0.030
Stanford AD classification			
Type A	11 (36.7%)	10 (76.9%)	0.015
Type B	19 (63.3%)	3 (23.1%)	
Aortic pathology			
Acute	8 (26.7%)	9 (69.2%)	0.009
Chronic	22 (73.3%)	4 (30.8%)	
Three-channel AD	2 (6.7%)	none	0.518
Family history of AD	17 (56.7%)	3 (23.1%)	0.043
Other coexisting conditions			
Hypertension	16 (53.3%)	8 (61.5%)	0.870
Hyperlipidemia	1 (3.3%)	2 (15.4%)	0.440
Diabetes mellitus	none	1 (7.7%)	0.677
CKD (Cr ≥ 2.0)	none	2 (15.4%)	0.028
Smoker	9 (30.0%)	5 (38.5%)	0.850

*CTD* genetically diagnosed connective tissue disease, *AD* aortic dissection, *CKD* chronic kidney disease, *Cr* creatinine value, *NS* no significant difference

In these series, the surgical treatments were performed according to the same indication criteria at the single center with the same surgical team staff. In the acute phase, emergent or urgent surgeries were carried out, in cases with acute type A aortic dissection with patent false channels excluding intramural hematoma, and with complicated acute type B aortic dissection. In patients with chronic aortic dissection or with residual aortic dissection in the long term after the initial surgical replacement, surgical interventions were indicated when the aortic maximum diameter exceeded 50 to 55 mm. Root repairs such as aortic valve-sparing surgery or composite valve-graft root replacement were carried out with the site of the root over 40 to 45 mm, predominantly for the CTD patients [6]. In 2 patients described above, surgical treatments were indicated for 3-channel dissection, even though the diameter was less than 50 mm.

### Histopathological examination

**Fig. 1:**
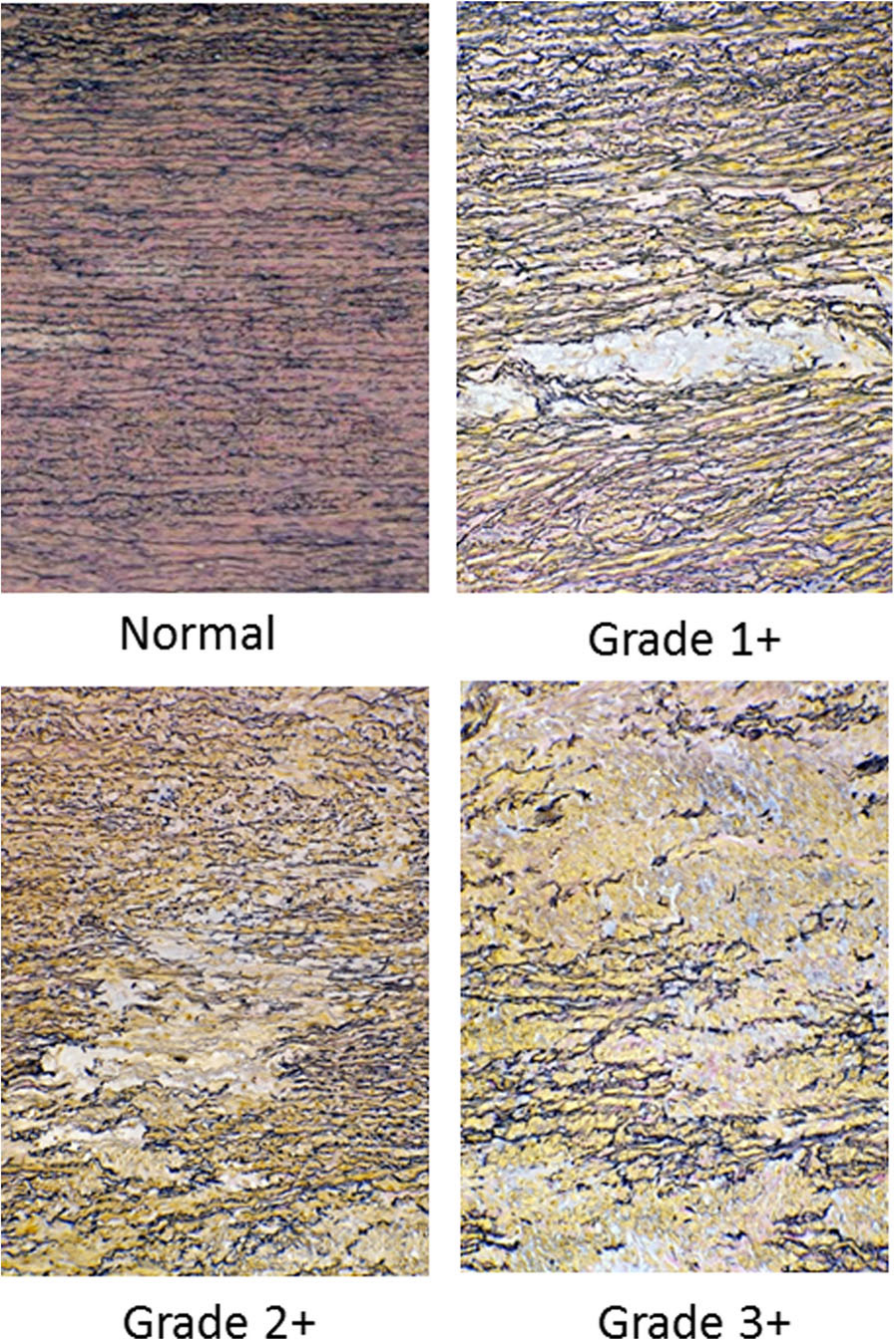
Elastin fragmentation

The samples were surgically resected from the dissecting aortic wall. Histopathological examination consisted of light microscopy on the surgical specimen stained with hematoxylin-eosin stain, Masson’s trichrome stain, elastica van Gieson stain, and toluidine blue stain. CMN was diagnosed if the aorta displayed fragmentation of elastic fibers associated with cystic accumulation of the extracellular matrix. The degree of loss of elastic fibers was defined as elastin fragmentation in the media stained with elastica van Gieson using a diagram of the grading system. Three grades were recognized as follows: grade 0 or 1+ (no loss or minimal loss or mild fragmentation of elastic fibers), grade 2+ (intermediate change), and grade 3+ (complete loss of elastic fibers in some full-thickness portions of the media) (Fig. 1) [5]. Afterwards, loss of elastic fibers was combined into two clinically relevant categories, namely, (1) no or minimal loss of elastic fibers (grade 0 or 1+ representing no loss or minimal loss or mild fragmentation of elastic fibers), and (2) loss of elastic fibers (grade 2+ or 3+ representing a significant loss of elastic fibers in some portion of the media) [5, 7].

### Genetic examination (sequencing and mutation analysis)

Mutation analyses were performed by bidirectional Sanger sequencing of exons and exon-intron boundaries of *FBN1*, *TGFBR1*, and *TGFBR2* genes first [8–11]. PCR products were purified and sequenced using BigDye Terminator chemistry v.1.1 on an ABI Prism3130xl or 3730xl (Applied Biosystems). In cases in which mutations of *FBN1* gene were not found, mutations were further examined with the multiple ligation probe amplification method on an ABI Prism3130xl (Applied Biosystems). In cases in which these two methods failed to find mutations in patients, *SMAD3*, *ACTA2*, and *TGFB2* genes were additionally analyzed by bidirectional Sanger sequencing of exons and exon-intron boundaries. For patients without mutations in *FBN1*, *TGFBR1*, *TGFBR2*, *SMAD3*, *ACTA2*, or *TGFB2* genes, exome sequencing was performed after TruSeq Exome enrichment on HiSeq1000 (Illumina) for searching mutations in *MYH11*, *COL3A1*, *COLIAI (COL1A1)*, and *COL1A2* genes [12–16]. Nonsense, missense, or splicing variations in these genes were further analyzed by Sanger sequencing if they were not present in SNP databases, predicted to be damaging by PolyPhen-2, or the SIFT program, or previously reported to be a pathogenic mutation.

### Follow-up

Data were collected from hospital admission and outpatient medical records. All patients were regularly followed, either at our center or at other local hospitals. All of them underwent strict blood pressure control before leaving the hospital, with β-blockers in all cases and angiotensin II receptor blockers in some cases. The follow-up rate was 100% and the duration was 57.1 ± 43.0 (range, 6–132) months.

### Statistical analysis

Two-tailed Student’s *t* tests for continuous variables and chi-square tests for categorical variables were used to make univariate comparisons between the two groups. The long-term outcomes including re-dissection and re-operation were analyzed using Kaplan-Meier methods and compared with log-rank tests. The risk factors (see Appendix) for mortality and late aortic re-operations were estimated using a multivariate proportional hazard regression analysis (Cox model). A *p* value of <0.05 was considered statistically significant. SPSS software, version 22.0 for Windows (IBM SPSS Inc., Chicago, IL) was used for all calculations.

In this study, between the CTD and the non-CTD groups, the early and late outcomes of the initial aortic surgery for aortic dissection were compared to demonstrate the impact of CTD on the surgical outcomes in the patients with aortic dissection associated with CMN.

## Results

### Initial surgeries for aortic dissection

As shown in Table 4, in the CTD group, there were initial surgical repairs of the ascending aorta in 5 patients and the aortic arch in 9 patients. Simultaneous aortic root repairs were performed in 11 of these 14 patients. The others underwent repairs of the descending aorta (in 10 patients), the thoracoabdominal aorta (in 2 patients), and the abdominal aorta (in 4 patients). In the non-CTD group, the surgical repairs were for the ascending aorta in 3 patients and the aortic arch in 7 patients. Simultaneous aortic root repair was carried out in 1 patient. The other 3 patients underwent repairs of the descending aorta (in 1 patient) and the thoracoabdominal aorta (in 2 patients). There were concomitant mitral valve surgeries in 2 patients in the CTD group.

**Table 4:** Initial surgery

Initial surgery (n = 43)	CTD (n = 30)	non-CTD (n = 13)	*p* value
Surgical site of aorta			
Ascending aorta	5 (16.7%)	3 (23.1%)	0.944
Aortic arch	9 (30.0%)	7 (53.8%)	0.253
Descending aorta	10 (33.3%)	1 (7.7%)	0.164
Thoraco-abdominal aorta	2 (6.7%)	2 (15.4%)	0.740
Abdominal aorta	4 (13.3%)	none	0.417
Simultaneous aortic root	11 (36.7%)	1 (7.7%)	0.115
Concomitant mitral valve surgery	2 (6.7%)	none	0.869
Conditions of false channel after the initial surgery			
Double barrel	23 (76.7%)	8 (61.5%)	0.519
Partial thrombosed	2 (6.7%)	2 (15.4%)	0.740
Thrombosed (IMH)	5 (16.7%)	3 (23.1%)	0.945

*CTD* genetically diagnosed connective tissue disease, *NS* no significant difference, *IMH* intramural hematoma

### Histopathological findings

CMN classification showed minimal or no loss of elastic fibers in 3 patients (10%) and loss of elastic fibers in 27 patients (90%) of 30 CTD patients, whereas minimal or no loss of elastic fibers was found in 6 patients (46.2%) and loss of elastic fibers in 7 patients (53.8%) of 13 non-CTD patients. The rate of loss of elastic fibers was significantly higher in the CTD group (*p* = 0.014).

### Postoperative data

**Fig. 2:**
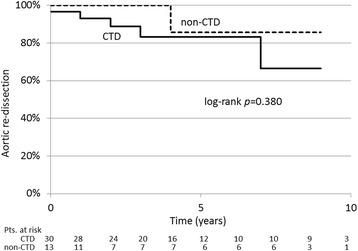
Absence of aortic re-dissection (Kaplan-Meier). (CTD = genetically diagnosed connective tissue disease)

There were no early deaths. In the long term, despite of there being no late deaths, new aortic dissection occurred at the same sites (three-channel aortic dissection) in 3 patients or at different sites in 3 patients (a total of 6 patients [20.0%] of the CTD group), whereas only 1 patient (7.7%) in the non-CTD group developed it at a different site (*p* = 0.310). The absence of re-dissection was not significantly different between the two groups (log-rank, *p* = 0.380) (Fig. 2), that is, 83.2% in the CTD group and 85.7% in the non-CTD at 5 years.

In contrast, in all, there were no significant differences in the incidences of re-dissection and re-operation, when all of the patients were divided into the two patient groups according to the grade of CMN. Of 43 patients, 9 patients had no or minimal loss of elastic fibers (Grade 1+) and 34 patients had loss of elastic fibers (Grade 2+ or 3+). There were no occurrences of re-dissection and 5 (55.6%) patients required re-operation in the former group, whereas 7 patients (20.6%) developed re-dissection and 25 patients (73.5%) required re-operation in the latter group. However, interestingly, all 7 patients who suffered from aortic re-dissection had high-grade CMN, that is, loss of elastic fibers (Grade 2+ or 3+); these 7 patients consisted of 6 (22.2%) of 27 CTD patients and 1 (14.7%) of 7 non-CTD patients (*p* = 0.951).

**Fig. 3:**
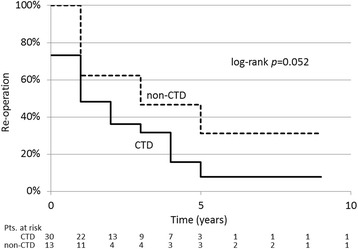
Absence of aortic re-operation (Kaplan-Meier). (CTD = genetically diagnosed connective tissue disease)

There were no significant differences in the re-operation rates between the Stanford aortic dissection classification groups (*p* = 0.332), that is, 13 of 21 with type A (62.0%) and 17 of 22 with type B (77.3%). There were also no significant differences in the absence of aortic re-operation (log-rank, *p* = 0.404), that is, 13.8% with type A and 14.5% with type B at 5 years. In contrast, re-operations were required more frequently (*p* = 0.037); there were 24 (80.0%) patients in the CTD group versus 6 patients (46.0%) in the non-CTD group. *The absence of aortic reoperation tended to be worse* (log-rank, *p* = 0.052), that is, 7.9% in the CTD group and 31.0% in the non-CTD group at 5 years (Fig. 3). *Twelve of 24 CTD patients (50.0%) and 3 of 6 non-CTD patients (50.0%) required thirdly surgical interventions.* Despite no substantial differences in the initial and following surgical procedures between the two patient groups, the rate of surgical interventions to the descending aorta was significantly higher in the CTD group (*p* = 0.021), that is, 16 patients (53.3%) versus 2 patients (15.4%). The rate of surgical interventions to the aortic root was also significantly higher in the CTD group (*p* = 0.037), that is, 15 patients (50.0%) versus 2 patients (15.4%). Regarding the time frame for aortic re-interventions, 15 of 24 patients (62.5%) in the CTD group underwent re-operations during the two years of follow-up after the initial surgery, compared with 3 of 6 patients (50.0%) in the non-CTD group. Univariate analysis revealed that patent false channel (*p* = 0.019), and simultaneous aortic root repair (*p* = 0.024) were significant risk factors for re-operation. In the multivariate analysis of risk factors for re-operation, patent false channel, simultaneous aortic root repair, and preoperative systemic hypertension were independent risk factors (Table 5).

**Table 5:** Multivariate analysis of risk foctors for re-operation

Variables	HR	95% CI	*p* value
Patient-related risk factors			
Hypertension	3.45	1.12–10.63	0.031
Dissection-related risk factor			
Patent false channel	5.22	1.37–19.89	0.016
Procedure-related risk factor			
Simultaneous aortic root repair	4.31	1.37–13.60	0.013

*HR* hazard ratio, *CI* confidence i

## Discussion

CMN is often observed in the resected aorta in patients with CTD and tends to be associated with the worst long-term prognoses; this also causes aortic dilatation and dissection in non-CTD patients as well as in CTD patients [1, 17, 18]. However, it is difficult to demonstrate pathologically remarkable differences in the fragmentation of elastic fibers and accumulation of extracellular matrix in the formation of the cystic structures between CTD and non-CTD patients, especially in elderly patients with systemic hypertension [4, 5]. In this study, we hypothesized that coexistence of genetically identified CTD would be associated with worse postoperative prognoses after surgeries for aortic dissection in patients with CMN. To elucidate it, we compared early and late outcomes, including occurrence of new aortic events after the initial surgeries for aortic dissection of CMN-positive patients, between the CTD and non-CTD groups.

CMN might be due to long-term hemodynamic forces (hypertension) and age [4, 5]. It was demonstrated that patients with CMN have increased risks of serious vascular complications [19, 20]. However, few studies have seemed to look at adverse effects of CMN on the surgical outcomes of aortic dissection, as far as we investigated. With regard to findings of histological examinations, Trotter and Olsen reported that the degrees of elastic fragmentation were variable in Marfan patients, and can also be found even in non-Marfan subjects [4]. Nakajima et al. pointed out a higher degree of elastic fiber loss or fragmentation in Marfan patients, compared with non-Marfan patients having CMN [21]. Similarly, in our study, the grade of loss or fragmentation of elastic fibers was higher in the CTD patients than that of the non-CTD patients. Clinically, in this study, the age of the onset of initial aortic dissection was also significantly younger in the CTD patients, and aortic re-dissection occurred only in the patients with high-grade CMN, that is, loss of elastic fibers. Such clinical phenomena may be affected directly by such fragility of the aortic walls due to higher degree of loss or fragmentation of elastic fibers in the aortic wall. Consequently, the presence of coexisting CTD should be suspected or examined genetically in relatively young patients with higher grades of loss or fragmentation of elastic fibers in the aortic wall.

With regard to occurrence of re-dissection in the long term after the initial aortic dissection, the incidence was higher in CTD patients, despite no significant statistical differences. Interestingly, in the CTD group, 2 patients had suffered from three-channel dissection before the initial surgery and another 3 patients developed it in the long term after surgery; this may show a more severe fragility of the degenerative aortic wall in CTD patients. In the histopathological examinations, the grades of loss or fragmentation of elastic fibers were higher in CTD patients than in those of the non-CTD group. Looking at the relationship between the histopathological findings and the re-dissection rate, there were no significant differences between patients with no or minimal loss of elastic fibers and those with loss of elastic fibers. However, interestingly, all of the CTD and non-CTD patients suffering from aortic re-dissection had high-grade CMN, that is, loss of elastic fibers in the histopathological examinations. Consequently, it is important to estimate the long-term outcome after initial surgery for aortic dissection according to the results of genetic examinations as well as the histopathological findings.

In contrast, regarding re-operations, in CTD patients, higher rates of redo surgery were reported than in non-CTD patients [22, 23]. In our study, redo surgeries were also required more frequently in CTD patients. In particular, the rate of descending aortic repairs was significantly higher, compared with non-CTD patients. In patients with CMN combined with CTD, higher onset rates of type B aortic dissection were demonstrated than the others [24]. Our study also demonstrated the higher rate of type B aortic dissection in CTD patients. Related to this issue, Schoenhoff et al. reported that 86% of Marfan patients with type B aortic dissection required redo surgery for residual aortic enlargement [25]. In our study, similar findings were revealed. In the CTD group, 80.0% of the patients required re-operations after the initial surgical repairs in the long term, 72.7% in type A and 84.2% in type B.

The rate of surgical interventions to the aortic root was also significantly higher in the CTD group. Progressive enlargement of the aortic root is one of the characteristics of CTD patients, which is related directly to the early onset of type A aortic dissection. In cases with aortic root enlargement of 40 to 50 mm in diameter, prophylactic root repairs such as aortic valve-sparing surgery or composite valve-graft root replacement are recommended in the guidelines to eliminate risks of ruptured type A aortic dissection [26]. Progressive aortic root enlargement also should be one of the reasons for redo surgery after various surgical repairs for type A or B aortic dissection, whether it is associated with aortic dissection or not. In our study, a total of 39.5% of all patients underwent aortic root repairs; this included 63.6% of CTD patients and 20.0% of non-CTD patients with type A aortic dissection, and 42.1% of CTD patients with type B aortic dissection. Moreover, simultaneous aortic root repair was a significant risk factor for re-operation in the univariate and multivariate analyses. Consequently, these circumstances resulted in the higher rates of redo surgeries in the CTD patients.

Obviously, there are some limitations to this study. First, this is a retrospective study dealing with a small number of enrolled patients. In particular, the number of the patients undergoing genetic examinations was too small to elucidate exactly the impact of CTD on the outcomes of surgeries for aortic dissection. Aortic dissection was also variable, such as acute or chronic, and type A or B. Apart from the histological aortic pathologies, there are other factors or requirements for redo surgeries after initial surgeries for aortic dissection, relating to types of aortic dissection, conditions of the false channels, surgical techniques, extent of repairs, and so on. The surgical procedures of the initial operations for aortic dissection were variable, which depended on a variety of conditions, including the stages of aortic dissection, such as acute and chronic, and the settings of the surgery, such as emergent/urgent and elective. Theoretically, the re-operation rates become significantly higher in cases with no tear resection and patent false channel [27, 28]. Actually, there may be some differences in the incidences of re-operation between limited ascending/hemiarch replacement and entire arch replacement. The rates should also be different after the total arch replacement between with or without elephant trunk procedures on the distal anastomosis site [29]. The conditions of aortic dissection, including the patency of the false channel, were also variable. More detailed studies dealing with a larger number of patients is required to elucidate precisely the impact of genetically identified CTD on the surgical outcomes of aortic dissection in patients with CMN.

Finally, most (90.0%) of the CTD patients showed high-grade CMN, that is, loss of elastic fibers. However, similar findings were also found in a half (53.8%) of non-CTD patients. The genetic examinations are still limited by the issue of availability. There might be other different CTDs with unknown genetic characteristics. Consequently, in case of high-grade CMN, it is necessary to recognize, even without genetically diagnosed CTD at present, potentially high-risk subjects who would develop re-dissection and require re-operations at relatively shorter intervals after surgery for aortic dissection.

## Conclusions

Patients with CTD as well as CMN in the aortic wall tend to develop aortic dissection, particularly of type B, at an earlier age. They require re-operation more frequently due to enlargement of the residual dissecting aorta and/or the aortic root, compared with non-CTD patients. Therefore, closer and stricter follow-up with medication and with earlier and more adequate surgical treatments including more extended repairs should be considered for such high-risk patients having CTD and CMN, and for some non-CTD patients having high-grade CMN.

## Abbreviations

CMN: Cystic medial necrosis
CTD: Connective tissue disease
